# Organizational and Individual Outcomes of Health Promotion Strategies—A Review of Empirical Research

**DOI:** 10.3390/ijerph18020383

**Published:** 2021-01-06

**Authors:** Agata Basińska-Zych, Agnieszka Springer

**Affiliations:** Department of Finance and Banking, WSB University in Poznań, 61-895 Poznań, Poland; agnieszka.springer@wsb.poznan.pl

**Keywords:** health promotion, intervention, organizational strategies, outcomes, workplace, enterprises, workers’ health, wellbeing, health promotion management, effectiveness evaluation

## Abstract

The main purpose of the paper is to identify the outcomes for employers and employees indicated in research related to workplace health promotion interventions (WHPIs). We investigated what methods are used and what types of organization this type of research is most often carried out in. In addition, the authors attempted to assess to what extent the methods used in the previous research prove the effectiveness of the implemented WHPIs. A systematic review of English-language papers (2000–2020) focused on types of health-promoting interventions in the workplace, and outcomes for employers and employees were conducted using the SCOPUS database (*n =* 260). As a result, 29 texts qualified for a final qualitative synthesis of the results. The analyses were most frequently conducted in small and medium-sized enterprises (SMEs) based on both quantitative and qualitative methods. In order to draw conclusions, analyses were made by classifying the research presented in the texts according to the type of intervention implemented, classifying the outcomes identified, and indicating the type of evaluation made by the researcher. The analysis showed that most of the outcomes presented refer to changes in the strategy and organizational culture, as well as the behavior of employees. In 18 studies, the indication of outcomes resulted directly from the evaluation outcomes. In other cases, the outcomes were identified by an evaluation of the process or structure of WHPI. The conducted analysis showed significant diversity in terms of the outcomes measured and the research methods used. The quasi-experimental methods, randomly controlled cluster trials, or cross-sectorial studies used in the study to confirm the effectiveness of WHPI were used only in every third study. In these studies, measurements were usually performed twice: at baseline and after intervention. The majority of studies confirmed that WHPIs led to a positive change in the healthy behavior of employees and effected an organizational change, and more rarely led to savings or a reduction in costs resulting from sickness absenteeism, presentism, turnover, etc., and return on investment (ROI). The article shows the need to conduct further research towards the development of guidelines for the evaluation of the effectiveness of implemented programs.

## 1. Introduction

For many adults, health is one of the most precious values [1]. Increasing life expectancy and the quality of medical services, as well as easy access to information on health, increases the level of public expectations when it comes to the quality of life. Fighting the negative effects of aging, taking care of one’s physical condition and appearance, as well as a holistic approach to health and mental strength are global values that play an increasingly important role [2]. These values also have a significant impact on professional life. An active lifestyle is not only a remedy for diseases of civilization, but also has an impact on limiting presentism, sickness absenteeism, falling productivity, and employee involvement. As Ilona Kickbusch notices, health is becoming increasingly important in various social areas [3,4]. However, health is defined by the WHO as “a state of complete physical, mental and social well-being and not merely the absence of disease or infirmity” [5] and, importantly, is determined to a large extent by the conditions in which the individual functions [6]. One of the most important environments that has an impact on health is work. OECD data show that workers in member countries spend approximately 1734 h per year at work, and although some countries report a slight downward trend, this still means that 36 h per week are spent at work on average [7]. The above situation means that people spend more active time at work than anywhere else. Objectively performed work in different professions can be a cause of accidents and occupational diseases [8,9,10,11], but it is also important for the general wellbeing of the individual, and the satisfaction and quality of work affects the satisfaction and quality of life outside the workplace [12,13,14].

For this reason, the activity of employers in the field of workers’ health cannot be limited only to actions resulting from legal bonds, e.g., in the area of ensuring safe and hygienic conditions, minimizing the risk of occupational diseases, and preventive examinations of occupational medicine. There are many terms and concepts that may be useful in describing a health effort that emphasizes voluntary activity and the investment approach of employers. These include health promotion at work—derived from the health sector—wellbeing at work [15,16], corporate wellness [17,18], management protection and safety culture, psychosocial determinants of health—including psychosocial risk—stress [19], or work–life balance, which are closer to the sectors of occupational safety and human resource management. They have a great deal of elements in common and often differ only in their distribution accents [20].

According to the declaration of the *European Network for Workplace Health Promotion*, “Workplace Health Promotion (WHP) is the combined efforts of employers, employees and society to improve the health and wellbeing of people at work. Health promotion includes activities such as health education, disease prevention and local health policy. This can be achieved through a combination of: improving the work organization and the working environment, promoting active participation, [and] encouraging personal development” [21]. Health promotion includes the reduction of risk factors and engaging in health-promoting activities [22].

Legal regulations, public expectations, the activities of the WHO and other health promotion organizations, along with the increasing openness of establishments to the needs of workers, have led an increasing number of organizations to introduce health promotion interventions [22]. Workplace health promotion intervention (WHPI) is a combination of program elements or strategies designed to induce behavioral changes or to improve the health status of individuals or the population as a whole. As Goetzel and colleagues note, the expectations of employers as to the outcomes of the implemented WHP are varied and optimistic [23]. However, companies are not always able to assess whether an implemented WHP has actually worked. Although in practice there are descriptions of many successful implementations of workplace health promotion programs (WHPP) [24,25,26], which indicate numerous organizational and individual benefits, an increasing number of systematic reviews conclude that there is not enough consistent evidence to reach a conclusion about the effectiveness of organizational interventions for health promotion [27,28,29]. The evaluation of effectiveness depends on the type of intervention [30,31], the measurement methodology [31] and implementation strategies, settings, or integration with local practice [32], and empowerment at the workplace. At the same time, in many cases, the effectiveness of the implemented programs is not tested at all or is tested selectively. The fulfilment of all methodological requirements related to the selection of a sample, the measurement of disruptive factors, or the need to repeat the measurement several times [33] means that, in many cases, the measurement of effectiveness is limited.

Therefore, the authors decided to focus firstly on recognizing the type of outcomes reported in various enterprises and on the methods of their measurement used in earlier studies. Secondly, the authors attempt to analyze what kind of program evaluation was carried out and whether there was any hard evidence of its effectiveness. The main purpose of the paper is to identify the outcomes for employers and employees indicated in the research related to workplace health promotion interventions. In addition, the authors attempted to assess to what extent the methods used in the previous research prove the effectiveness of the implemented WHPIs. The following exploratory research questions were asked in the current article:

- Q1: In what types of enterprises are the outcomes of WHPI examined and measured most often?
- Q2: What research methods are used to measure the outcomes of WHPI?
- Q3: What types of outcomes of WHPI are reported by organizations?
- Q4: How often has research indicated strong evidence data confirming the effectiveness of WHPI?

## 2. Literature Review

Van der Vliet defines health at work as a set of a number of activities undertaken by different health professionals to implement a strategy aimed at maintaining healthy and safe working conditions [34]. At the same time, activities aimed at creating a healthy organization include both individual health-promoting practices and organizational conditions, which consist of a company culture, leadership principles, and values. Moreover, activities undertaken by organizations must be consistent with strategies undertaken by local or international organizations, such as the European Network for Workplace Health Promotion [35]. WHP is described as the process of enabling employees to increase control over their health and creating not only a way to increase awareness of health determinants, but also supporting them in changing their lifestyle [36]. Thus, improving the wellbeing of workers is one of the outcomes of WHP, as is wellness. From this perspective, WHP focuses on better health outcomes through measurable improvements beyond merely reducing short-term risk or addressing direct health threats [37]. Health promotion outcomes are changes in the personal characteristics and skills of workers and/or social norms and activities and/or organizational practices that can be attributed to health promotion activities [38]. The health and wellbeing of employees is a result of a balance of physical, mental, and social components, as well as health habits related to good physical condition, energy, and vitality.

While researching the links between workers’ health and productivity, the importance of improving workplace ergonomics [39,40], working conditions [41,42], and organizational change [43] or the impact of physical activity [44] are also of key importance. However, clear empirical evidence combining the effectiveness of comprehensive health promotion strategies applied in enterprises with the improvement of employee health and performance and efficiency at the organizational level is still not available.

Health management at work is developing towards a multi-faceted approach that integrates activities in the area of health protection and promotion [45] as well as indicating the broad consequences of the actions taken, which not only contribute to improving the functioning of the employees or their organization but are also relevant to public health [46]. A model approach to the promotion of health at work is presented by Sorensen et al., who emphasize the importance of integration of activities, i.e., (1) leadership commitment, (2) coordinated efforts, (3) supportive organizational policies and practices, and (4) comprehensive program content [45]. At the same time, this model indicates three types of achievable results, two groups of results related to the benefits achieved by employees (proximal and further) and organizational results. The analysis devoted to the review of research of the analyzed scope will include all these three groups of results. In addition, we treat work conditions as organizational proximal outcomes and include them in the analysis.

It is worth noting that health promotion activities can be very diverse, and they can affect working conditions, ranging from environmental conditions and work organization, to psychological factors, to shaping the objectives and tasks entrusted to employees, as well as influencing (e.g., through educational programs) the attitude, knowledge, skills, and behavior of employees [47]. Health promotion interventions in the workplace may focus on just one (single-focused) or many (multi-focused) specific areas and objectives for the health of employees, such as: Reducing smoking and alcohol consumption among employees; promoting healthy eating at the workplace; increasing physical activity and improving fitness; reducing stress; and enhancing leadership and personal development. These activities can be addressed to individuals or to a group of workers with specific disorders, e.g., obesity, musculoskeletal disorders, mental health, and diseases, e.g., heart disease and strokes, diabetes, cancer, osteoporosis, and arthritis. Interventions are implemented through programs and projects.

The Center of Disease Control and Prevention defines WHPPs as a coordinated and comprehensive set of health promotion and protection strategies implemented at the worksite that includes programs, policies, benefits, environmental support, and links to the surrounding community designed to encourage the health and safety of all employees [48]. They should consist of the following components: (1) Health education programs, (2) a supportive social and physical environment, (3) integration of the program into the organizational structure, (4) screening, including treatment and follow-up as needed, and (5) links to other assistance programs [33].

Using different methods, such programs provide not only a great deal of diagnostic information about the health of employees, but also specific interventions aimed at identified risk factors related to prevention, promotion, and recovery [49]. Importantly, comprehensive WHPPs provide health education, and are linked to special services for employees, supporting changes in the physical and social work environment to improve health and organizational culture [50].

However, the question remains as to whether and to what extent the various programs are successful and effective. Successful interventions may be defined as actions that result in significant and sustainable behavior changes and translate behavior change research into real-world settings [51]. Industry reports such as “Working Well: A Global Survey of Workforce Wellbeing Strategies” indicate that organizations see the benefits of taking such actions which, according to the companies surveyed, contribute to positive changes in engagement (86%), organizational image (82%), overall wellbeing (78%), recruitment and retention (76%), and productivity (76%) [52]. However, researchers recognize that attempts to measure the effectiveness of health promotion research often face methodological problems [53,54]. As organizations often introduce measures for their own use that allow them to estimate the benefits of the implemented programs, these results are difficult to generalize to other entities due to the unitary arrangement of a number of relationships. The results of evaluating the effectiveness of WHPPs also often lead to misleading or wrong conclusions. Among the most frequently mentioned reasons for this fact include: The lack of application of a rigorous methodology of intervention evaluation; the high costs of evaluation, which in many cases exceed the intervention budget; or the pragmatic reasons justifying the continued existence of the program; or no evaluation plan [55]. However, measuring the progress and outcomes of WHPP is crucial to providing evidence of its effectiveness.

Referring to the Modified Worksite Health Promotion Logic Model (Assessment of Health Risk with Follow-Up) adopted by the CDC Community Guide Task Force, three levels of WHPP effectiveness evaluation, namely structure, process, and outcomes, are distinguished. Specifically, a structure evaluation is used to assess the “inputs” of the WHPP with the help of indicators that measure organizational policies, programs, and environmental support [33,56]. The second level of assessment refers to the process, which consists of metrics describing tangible results from WHPP such as participation rate, engagement level, awareness, and satisfaction of employees. The last set of elements in the model focuses on outcome evaluation, special health, psychological, or other variables that WHPP is designed to improve over time e.g., absenteeism, changes in cardiovascular risk, and perceived stress [33,56]. While focusing on program outcomes is the basis of a good and complex assessment of program effectiveness, it seems that evaluating the program without assessing the components of the structure and process is insufficient. The above model became the basis for the analysis of the collected material in terms of assigning the level of evaluation of implemented WHPP.

## 3. Materials and Methods

**Figure 1:**
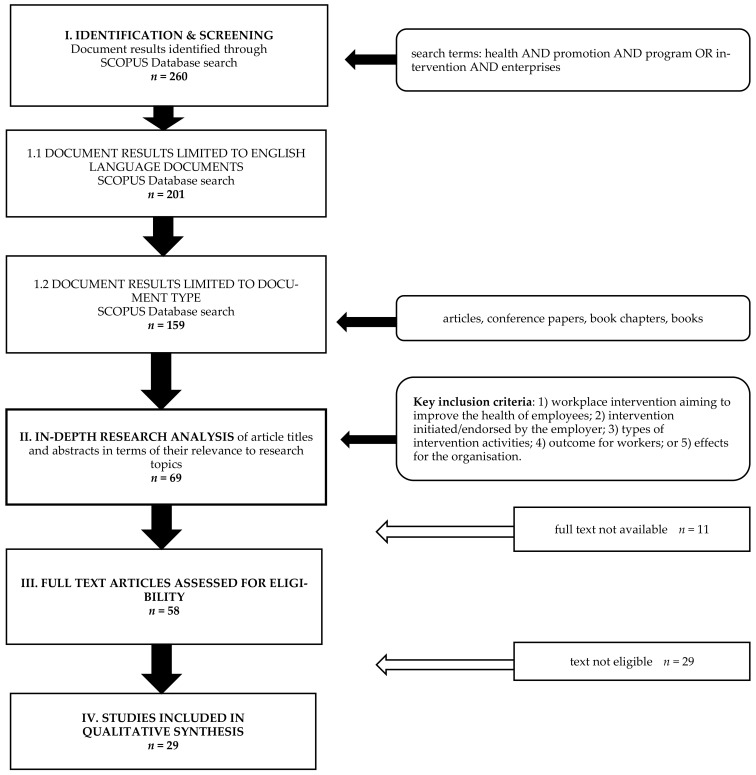
Flow diagram of systematic literature search. Source: Author’s compilation.

The scientific literature focusing on health promotion interventions and programs in enterprises and outcomes for employers and employees published in English between 2000 and March 2020 was systematically searched in the SCOPUS database [57], which has been selected to access research from different countries in a number of areas, in particular including peer-reviewed publications from the fields of medicine, health, business, management, and social sciences. A diagram of the research procedure and systematic literature review is presented in Figure 1. The sampling procedure for the study consisted of four stages: (1) Identification and screening (in Scopus), (2) in-depth research analysis of article titles and abstracts in terms of their relevance to research topics, (3) an analysis of the availability of full texts, and (4) an in-depth qualitative analysis of full texts [58]. In order to ensure the accuracy and consistency of the obtained data and the reliability of drawing conclusions from the analyzed studies, two scientists were independently engaged in all stages of the process.

In the first stage of the research, a variant of an advanced search in SCOPUS by elements of titles, abstracts, and keywords was selected, using the following terms in three lines: health AND promotion AND program OR intervention AND enterprises. The LIMIT-TO (LANGUAGE) “English” option was also used. The search results excluded the document type category: Review (44) and conference review (5), note (2), short survey (2), letter (1). Finally, 158 results were obtained in the form of: articles (80.4%), conference papers (13.3%), book chapters (5.7%), and books (0.6%).

**Figure 2:**
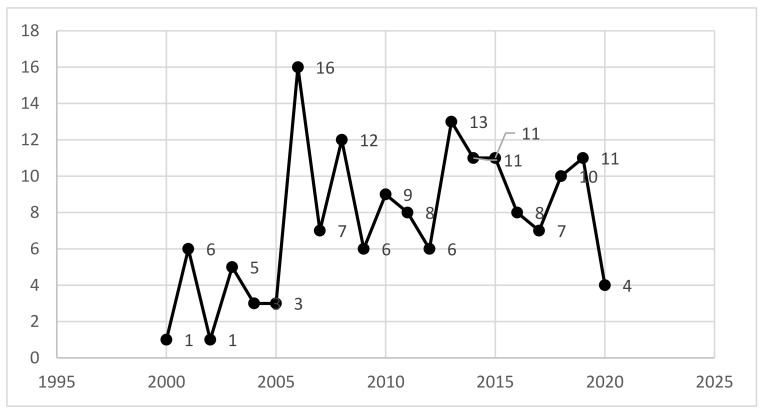
Results of searching in SCOPUS by year of document (*n =* 158). Source: Own work.

In total, publications originated from 43 countries. The largest number of texts searched for came from the year 2006 (16), followed by 2013 (13), 2008 (12), and 11 texts from 2014, 2015, and 2019 (Figure 2). We can observe growing scientific interest in the subject of pro-health interventions in enterprises, although as of March 2020, we have only four texts.

The second stage of the work consisted of an in-depth analysis of the results, including the titles and abstracts of the publications. Key inclusion criteria for in-depth research were: (1) Workplace intervention aiming to improve the health of employees; (2) intervention initiated/endorsed by the employer; (3) types of intervention activities; (4) outcomes for workers; or (5) effects for the organization. As a result, 69 publications qualified for the next stage of analysis. The next step was to examine the availability of full texts in various databases. At this stage, as many as 11 texts could not be accessed.

In the fourth stage of the in-depth analysis of the results, the full texts of 58 publications were subjected to the procedure. Twenty-nine publications were rejected because of the nature of the intervention concerning public health rather than enterprises, because of the implementation of the national health promotion program, because of a different recipient of the program than employees or enterprises (e.g., local community, people with disabilities), because of the overly narrative style of the article without details, or because the analysis focused on only one health and safety component in the workplace, such as return-to-work studies. Some of the rejected texts did not describe either the outcomes for employees or the results of the implemented intervention for the organization, or they did not include information describing the WHP intervention or described only the planned research. Each study that met the criteria for inclusion was assessed for the adequacy of intervention and outcome evaluation by at least two independent authors. Applying the inclusion criteria as in the fourth stage, 29 texts qualified for the final qualitative synthesis of the results.

Qualitative analysis of the material collected was performed according to the following steps:

1. The identification of size and type of organization and the country in which the research was conducted.
2. The analysis of type and duration of the WHPI.
3. The identification and classification of outcomes indicated in the tests. Based on Sorensen’s model (see Figure 2), two types of organizational and four types of individual categories of outcome were identified. The categories used are not disjunctive, which means that in one research paper, the same and different types of outcomes could be identified.
4. The identification of the research methodology used to present the results of WHPIs. In particular, the method, sample size, tools, and frequency of outcome measurement were considered.
5. Level of evaluation carried out: Process, structure, and outcomes were taken into consideration.
6. The analysis and evaluation of strong evidence data confirming the effectiveness of WHPIs was conducted based on nine criteria/questions:
  - Were quasi-experimental methods or randomly controlled cluster trials or cross-sectorial studies used in the study to confirm the effectiveness of WHPI?
  - Was the WHPI effectiveness measurement carried out at least twice (at baseline and after intervention)?
  - Was the WHPI effectiveness measurement repeated after a certain period of time after the end of the intervention to check the durability of its effects? After what period of time?
  - Were objectified research tools used in the study?
  - When assessing the effectiveness of the WHPI, was the structure or process examined in addition to the outcomes as well?
  - Was the WHPI effect to modify unhealthy habits and improve the risk profile of employees, especially the highest risk groups?
  - Was the WHPI effect an organizational change?
  - Did the WHPI result in financial benefits for the organization?

## 4. Results

### Description of Material Analyzed

After the application of inclusion and exclusion criteria, 29 publications were considered relevant and subjected to critical review, 89.7% of which were articles, while the rest were conference papers. Nearly 63.0% of the examined publications concerned the subject of medicine and health, 21.7% environmental studies, 13.1% social sciences, and 2.2% from another subject area. The most popular journals which the articles came from were Industrial Health (four articles), International Journal of Environmental Research and Public Health (three), Journal of Occupational Health (three), and Health Education Journal (two).

The collected research material was analyzed according to the following research criteria: (1) Year of publication, (2) type of paper, (3) research area (country); (4) size of organization; (5) sector; (6) type of research; (7) research subject and sample size (Table 1).

The majority of the studies that qualified for critical analysis were published in the period of 2011–2020 (69%), and the rest in 2000–2010 (31%). Out of 29 studies analyzed, the vast majority (89.7%) were carried out in one country (most often in Europe); only three studies were carried out in several countries. The final selection included 93.1% (27) original papers and 6.9% (2) review articles. Populations analyzed in original papers included a total sample of 35,355 employees and 1081 enterprises. Systematic reviews investigated a total of 107 original studies and a total sample of 54 interventions and 4 projects lasting several years.

- Q1: In What Types of Enterprises are the Outcomes of WHPI Examined and Measured Most Often?

Taking the responses to Q1 into account, small and medium enterprises (SMEs) were the most numerously represented (44.8%) type of entities in which outcomes of WHPI were measured. Moreover, 17.2% of studies were carried out in large companies, 13.8% only in small companies, and 3.4% in medium-sized companies. Almost 10% of such work was carried out in companies of all sizes, while 10% of the studies did not indicate the size of the investigated entity (Table 1). In addition, 36.7% (eight) of the analyzed studies concerned research in various sectors. In 16.7% of studies, researchers focused on manufacturing and industry enterprises, services or retail entities, or other sectors, such as informal sectors, social enterprises or public providers, or social care entities. Unfortunately, in 13.3% of studies, the sector had not been defined.

- Q2: What Research Methods are Used to Measure the Outcomes of WHPI?

With reference to Q2, methods of measuring the outcomes of work health promotion programs in enterprises were identified (Table 2). In 34.5% of the studies, the methodology of qualitative research was used, while 24.1% were based on quantitative research, and in 41.4% of the investigated studies, a mixed methodology was applied, often based on the triangulation of research methods. In this category, the quasi-experiment method, a survey or cluster randomized controlled trial, or cross-sectional studies were particularly popular. In addition to the survey questionnaires, researchers often used the WHPI evaluation reports, and other more objective research tools, such as medical check-ups, sickness absenteeism statistics, number of occupational accidents, thermal conditions of work, etc.

Qualitative studies were mostly based on case studies or conducted interviews and follow-up evaluation. The most popular research tools used in these qualitative studies were semi-structured interview questionnaires addressed to employees or employers, or protocols from focus groups consisting of representatives of a professional group. In contrast, quantitative methods included longitudinal test-retest study.

Moreover, the sample size is also a relevant issue. In 69.0% (20) of the studies, the sample size of investigated enterprises was indicated; in nine of them, the number of enterprises researched did not exceed 10, in eight of them the number ranged from 10 to 100, and three studies included an analysis of more than 100 enterprises. In 48.3% (14) of the studies, the number of employees surveyed was indicated; in three cases, the number did not exceed 100, in six of them, the number ranged from 100–999, and in five studies, the research covered more than 1000 employees. In 20.7% (six) of the studies, information on sample size was given for both enterprises and employees. Furthermore, the frequency of measurement was analyzed in previous studies (Table 3). In 27.6% (eight) of the studies, the measurement was conducted twice (pre- and post-test); in 17.3% of cases (five)—three times: At baseline, after implementation, and one year after implementation; and in 3.4% of studies (one study)—only once (after intervention).

- Q3: What Types of Outcomes of WHPI Are Reported by Organizations?

In accordance with Q3, the types of outcomes of WHPIs reported by organizations were analyzed. In more than half of studies, both organizational and individual outcomes of WHPI were indicated (58.6%). Nearly 27.6% of studies reported outcomes for employers, and 13.8% reported only outcomes for employees. Due to the high level of heterogeneity of the outcomes indicated in studies by other authors, the decision was made to categorize the outcomes of WHPIs in six major types: Organizational outcomes—namely culture and company strategy, and financial outcomes; and individual outcomes—behavioral, cognitive, psychological, and physiological (Table 2). The elements included in the conceptual model for integrated approaches by Sorensen et al. (2016) were the basis for the categorization of outcomes of WHPI.

**Figure 3:**
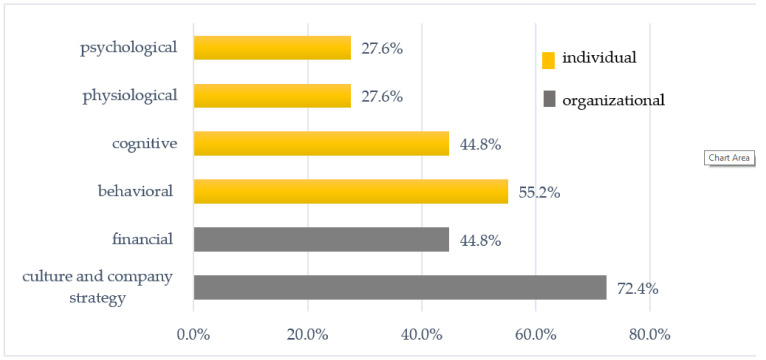
Organizational and individual outcomes of health promotion programs in the entities studied. Source: Author’s compilation.

In 72.4% of studies, outcomes related to organizational culture and company strategy were indicated (Figure 3). Among those indicated were improvement of the working environment and occupational climate, the creation of a workplace free of smoke or excessive noise, and the improvement in health awareness of companies or reinforcement of teamwork. Approximately 45% of the investigated studies reported financial outcomes e.g., reduced sickness absenteeism, presentism, turnover, preventing early retirement, or increased productivity of employees.

As regards individual outcomes of WHPIs, the studies most often reported behavioral (55.2%) results, which referred to leading to changes in the behavior of employees towards a healthier lifestyle, helping them to adopt and maintain healthy behaviors (sleep, exercising, a healthy diet, coping with stress), and the reduction of health risk behaviors (e.g., smoking). Approximately 45% of the investigated studies reported cognitive outcomes focused on changes in the beliefs, knowledge, and skills of employees. Less frequently, physiological (27.6%) and psychological (27.6%) outcomes were indicated in previous studies.

Due to the wide variety of health promotion initiatives reported in the investigated studies, interventions in the workplace have been classified into five categories (Table 3): (1) Improving the environmental conditions and work safety, (2) health education and counseling, (3) coping with health problems and personal development, (4) physical activity and fitness intervention, (5) a multi-focused comprehensive program (WHPP). The research results indicate that interventions in enterprises usually take the form of multimodal and comprehensive programs promoting mental health, wellbeing, and wellness (41.4%), e.g., Work Improvement in Small Enterprises (WISE) or Total Work Health ^®^ (TWH), where all employees or special groups of employees, e.g., workers aged 60+, women or immigrants, and blue-collar workers are the recipients of the measures. Moreover, interventions based on improving the environmental conditions of the workplace and work safety were reported in 27.6% of studies. In addition, activities in the workplace focused on physical activity or fitness accounted for 20.7% of studies. The least frequently reported interventions were health education and counseling—17.2% of studies, and activities in the field of coping with health problems and personal development—13.8%. To sum up, in the previous studies, the analyzed outcomes of WHPIs reflect its character. Major characteristics of interventions/health promotion programs in the entities that were the subject of study are presented in Table 3.

Furthermore, the duration of interventions was analyzed, revealing that the time range of WHPIs in the analyzed studies varied significantly. In almost 31.0% of studies, intervention took up to 6 months (short-term intervention), in 20.7% between 1–2 years (medium-term intervention), and in 17.2% of studies, more than 3 years (long-term intervention). Almost one-third of studies indicated no data pertaining to the duration of WHPI.

- Q4: How Often Has Research Indicated Strong Evidence Data Confirming the Effectiveness of the WHPI?

Measuring the progress of WHPP is essential in order to provide evidence of its effectiveness. A relevant question from the point of view of measuring the effectiveness of WHPI seems to be Q4, about evidence-based data used in previous studies. The results of analysis are presented in Table 4. Review articles were excluded from the analysis at this stage. According to experts [33], the most reliable methods focused on evidence-based procedures for proving the effectiveness of health promotion programs are quasi-experimental methods, randomly controlled cluster trials, or cross-sectorial studies, which were used in only eight (29.6%) of the analyzed studies. Unfortunately, it is not always possible to use experimental or quasi-experimental methods in evaluating the effectiveness of WHPI due to high costs, strict procedures, or ethical reasons. In 17 cases (62.9%), objectified research tools were used in previous studies, e.g., medical check-ups, sickness absenteeism statistics, number of occupational accidents, thermal conditions of work, etc.

An important element of the effectiveness measurement procedure is the frequency of its measurement. It was assumed that the measurement of WHPI effectiveness should be carried out at least twice: At baseline and after intervention (pre- and post-test). This occurred in 12 studies (44.4%). Taking into account the durability of the effects of intervention, measuring the effects a short time after the end of the WHPI is recommended, on average from a few months to a year after intervention.

Only in five studies (18.5%) was the evaluation of effectiveness conducted within a timeframe of at least a few months up to one year after implementation. A crucial issue was also the evaluation approach. Outcome evaluation was identified in 14 papers (48.3%), the process approach in 7 cases (24.1%), and in 4 studies, structure and process evaluation was presented (13.8%). Only in four studies did researchers evaluate the structure, process, and outcomes of WHPIs.

Moreover, the next important evidence of the effectiveness of a program is its ability to modify unhealthy habits and improve the risk profile of all employees, especially the highest risk members. In 26 studies (96.3%), WHPIs led to a change in the behavior of employees, becoming healthier or more safer, or preventing high-risk diseases or injuries. The behavior change in these studies was measured as well, in an objective—quantitative, as subjective—qualitative manner. In 24 (88.8%) of the indicated studies, the WHPI effected an organizational change. Such organizational changes mainly concerned the implementation of a healthier work environment, work schedules, a better organizational climate, and a healthier culture.

Furthermore, literature proves that effective pro-health interventions are programs that lead to savings or a reduction in costs resulting from sickness absenteeism, presentism, turnover, etc., and potentially have a positive return on investment (ROI). In 11 studies (40.7%), certain financial benefits resulting from the implementation of WHPIs were indicated. The most popular outcomes were decreased levels of sickness absenteeism, improved productivity, and profitability of the company.

## 5. Discussion

Creating healthier workplaces is an important element of organizational strategies based on the health and wellbeing of companies around the world. Although there is no one single reliable and effective organizational strategy to promote health in enterprises, more benefits for employee wellbeing and organizations were achieved by comprehensive interventions endorsed by the employer in the form of broader health policies or programs, usually defined as health promotion at work or corporate wellness.

The main purpose of the paper was to identify the outcomes for employers and employees indicated in the research related to workplace health promotion interventions (WHPIs). We also checked which methods are used and in what types of organizations this type of research is carried out most often. Based on a systematic review, we confirmed the results of other authors’ research that WHPI vary considerably in size and composition, and they have evolved significantly over the past 30 years [58]. More than two-thirds of the texts analyzed indicated outcomes related to the implementation of the comprehensive program, while others indicated outcomes related to one or more selected areas of intervention. Except for interventions related to physical activity, there are marked tendencies to include a wide range of activities in the area of health promotion in the analyses. Such an approach is in line with the recommendations contained in the integrated model that contains the causal pathways through which work may influence health outcomes [45].

Evaluating the effectiveness of such programs requires the use of a complex evaluation that should contain the process, structure, and results [33]. Such an approach was found only in four of the papers analyzed. The majority of the research focused on identifying the outcomes of the implemented interventions or programs, apart from evaluations of process and structure. It is worth emphasizing, however, that in the case of identifying the outcomes of single-focused interventions, researchers more often not only identified the outcomes, but also measured the effectiveness of WHPIs. We can therefore see the dilemma of researchers who attempt to strike a delicate balance between trying to evaluate the entire process of health management programs and the detailed examination of the effectiveness of a specific action. Moreover, the existing research results indicate that singular interventions showed limited effectiveness. Multilevel and multicomponent interventions may combine these actions and aim for changes at individual, social, and physical environmental levels. Such interventions and policies may have the greatest potential for effectiveness, and thus they may be appealing to practitioners and funding bodies [85]. However, the high level of complexity of interventions and policies hinders the identification of the factors responsible for their success [86].

The variety of measurement methods used, as well as the different levels of evaluation applied, did not exclude the identification of positive outcomes of WHPIs. In the area of organizational changes, those relating to culture and strategy were the most frequently mentioned. This result is coherent with other studies that indicate a positive impact on safety culture [87]. The indication of financial outcomes was less frequent and was most often associated with the costs of absenteeism and presentism. The result was not a surprise. The other scientists research confirmed the financial effectiveness of WHPI using e.g., the ROI methodology as well., In order to correctly prove a positive rate of return on investment in a WHPP, at least three years’ time horizon is needed (healthcare cost savings) [33,88]. Absenteeism outcome is likely to evidence in 24 months [33]. However, at the same time, evidence-based effects on rates of cardiovascular disease can took many years. This tendency is noticeable also in results by the authors of this article.

Moreover, many factors spill over into financial outcomes, the identification of relations is difficult, not only in the case of WHPP. This problem has been a topic of discussion in the HRM area for decades [89,90], however there is plenty of evidence of a link between HRM practices and employee attitude and behavior [91]. The same situation may be observed in the field of WHPI. Behaviors and attitudes were very common outcomes measured by researchers. As the implemented interventions are connected to many different aspects of health such as cognitive and behavioral aspects, employee outcomes are highly diversified. It is also worth emphasizing that while the level of the methodology used to measure the results is often insufficient, the choice of the outcomes does not raise any objections. In each study, the identified outcomes fit the type of intervention analyzed.

The quasi-experimental methods or randomly controlled cluster trials or cross-sectorial studies used in the studies to confirm the effectiveness of WHPI were used only in eight of the analyzed studies, as the most reliable method based on hard evidence data. The results of the research are in line with the observations of other authors, which confirm that the non-experimental (observational) studies are the most widely used in health promotion evaluations, very often with one group, posttest only, or with control group with twice-measurement frequency (at baseline and after intervention) [23,54,92].

## 6. Conclusions

A large variety of methods in the studies analyzed, different quality of results, and differences in sample size made it difficult to draw very clear conclusions (heterogeneous response, participation rates, different interventions, and outcomes). This problem has been recognized not only in this article but also in many other systematic reviews [30,87,88]. There is an urgent need to develop a uniform methodology for assessing the effectiveness of WHPP programs within the SME sector, taking into account the size of the enterprise and the sector of activity. Further implementation research is needed in this area. In the authors’ opinion, the research quality assessment procedure used in the study may be helpful in preparing the evolution of the implemented program or intervention.

The conducted systematic review has certain limitations, the first of which is related to the database from which the material for the review was obtained. In case of the use of databases other than SCOPUS, both the number or article and the subject area could be different. The second limitation results from use of systematic review rather than meta-analysis. The method used permitted only a qualitative, and not a quantitative, description of the outcomes described by researchers. However, the use of a meta-analysis would require a significant limitation of the outcomes analyzed.

To sum up, the systematic review carried out in the article showed a wide range of individual and organizational results identified during the WHPI assessment. The analysis showed that the efficiency assessment is a complex process and can be carried out using various quantitative and qualitative methods. Despite the lack of a uniform approach to the evaluation of implemented programs, the presented studies show the importance of pro-health activities both for employees and organizations. The paper shows the need to conduct further research towards the development of guidelines for the evaluation of the effectiveness of implemented programs with particular emphasis on small- and medium-sized enterprises.

## Figures and Tables

**Table 1:** Characteristics of studies included in the systematic review.

Study	Type of Paper	Country	Size of Organization	Sector	Type of Research	Sample Size	
						Enterprises	Employees
[59]	O	Norway	not indicated	manufacturing	3		125
[60]	O	China	SMEs	manufacturing	3	60	1211
[61]	O	UK	SMEs	varied sectors	3	132	
[62]	O	Belgium	not indicated	social enterprise	2		196
[63]	O	USA	small	varied sectors	1	19	
[64]	R	Poland	large, medium, and small	varied sectors	1		84
[25]	O	USA	SMEs	services	1	4	
[45]	O	USA	SMEs, hospitals	varied sectors	1	3	not indicated
[65]	O	Taiwan	SMEs	financial	3	31	428
[66]	O	Taiwan	large, medium, and small	varied sectors	3	544	
[67]	O	Colombia	large	public providers, IT and communication services	3	1	6000
[68]	O	Korea	medium	metal company	3		211
[69]	R	UK	not indicated	varied sectors	1	3	
[70]	O	Vietnam	SMEs	varied sectors	3	20	
[71]	O	China	large	retail	2	9	2768
[72]	O	UK	SMEs	varied sectors	3	17	89
[73]	O	Japan	large	services	2		22429
[74]	O	Wales (UK)	SMEs	varied sectors	1	5	
[75]	O	Taiwan	SMEs	not indicated	2		133
[76]	O	Ireland	SMEs	Health and Social Care Trust	1	18	
[77]	O	Japan	large	not indicated	3		1070
[78]	O	Norway	large, medium, and small	not indicated	3	11	
[79]	O	Japan	SMEs	not indicated	1		80
[80]	O	Saudi Arabia	large	oil refineries	1	2	
[81]	O	Philippines, Thailand, Japan	small	informal sector	3	8	
[82]	O	Thailand	small	manufacturing	3	7	
[24]	O	Germany	SMEs	varied sectors	2	150	
[83]	O	Wales [UK]	SMEs	varied sectors	2	37	531
[84]	O	Philippines, Thailand, Laos, Pakistan, Vietnam	small	workers and farmers, home-based workers	3		not indicated

1—qualitative; 2—quantitative; 3—mixed; O—original paper, R—review article, SMEs are small and medium-sized enterprises; Source: author’s compilation, *n* = 29.

**Table 2:** Categories of organizational and individual outcomes with characteristics based on the systematic review.

	Categories Used in Model Developed by Sorensen	Applied Categories of Outcomes	Outcomes Indicated in the Analyzed Studies
organizational	Conditions of work (physical environment, organization of work)	Culture and company strategy	Led to implementing a strategy and culture based on health, improved working environment and occupational climate, improved work organization, greater flexibility of the work schedule, creating a workplace free of smoke and excessive noise, improved health awareness of companies, improved CSR
	Enterprise Outcomes (productivity and quality, turnover and absence, health care costs)	Financial	Led to cost reduction in the long term, reduced sickness absenteeism, active sickness absence, employee expenditure on health care and better health intervention return on investment, reducing medical cost, positive impact on insurance absenteeism, presentism, turnover, preventing early retirement, increased productivity of employees, faster return to work in case of injury, and faster return after disability pension, as well as reducing the frequency of occupational accidents.
individual	Worker proximal outcomes (health and safety behaviors engagement in programs)	Behavioral	Led to reinforcement of teamwork, satisfaction from participating in the program, changed behavior of workers and managers, helping workers to adopt and maintain healthy behavior, lower health risks, improved healthy lifestyle of workers: healthy eating, less stress, more sleep, giving up smoking, physical activity or fitness classes, exercise, improved coping strategies
	Worker proximal outcomes	Cognitive	Led to enlightenment in terms of health awareness in the workplace, shared information about health issues, initiated thinking about health, improved ability to work, a sense of control over their jobs, and mobility to meet the mental demands of work, improved new skills and minimized skill underutilization
	Worker outcomes Injury Illness	Physiological	Led to an improvement in the physical health of workers, improvements in musculoskeletal disorders, pain reduction in neck, wrist pain and upper/lower back pain, chronic illness prevention, e.g., weight, blood pressure, resting heart rate, waistline, BMI, front and back trunk flexibility, abdominal muscle durability and back muscle strength
	Worker outcomes Wellbeing	Psychological	Led to improved mental health and wellbeing, reduction of stress-related hazards and consequences, a decreased level of occupational burnout, reduction of somatic symptoms, depressive symptoms, work-related symptoms, job satisfaction and lower job tension.

Source: author’s compilation, *n* = 29.

**Table 3:** Characteristics of workplace health promotion interventions (WHPIs) in the entities studied based on systematic review.

Studies	Characteristics of Intervention or Program	Classification—Type of Intervention Activities *	Duration of Intervention and Measurement Frequency	Type of Indicated Individual (I) and Organizational: (O) Outcomes *	Evaluation Types	Detailed Result of the Assessment of the Effectiveness of the Intervention **
[59]	A supervised high-intensity interval training intervention focused on cardiovascular health effects	physical activity & fitness intervention	3 years: 5 times (at baseline, after 3 months of intervention, after 1 year, after 2 and 3 years)	I: physiological; O: not applicable	outcome evaluation	cardiovascular health effects (+) (physical activity level as a moderator between work schedule and cardiovascular disease risk factors)
[60]	“Participatory Action-Oriented Training” (PAOT): the use of respiratory protective equipment, different types of intervention	environmental and safety workplace intervention; health education and personalized counseling	6 months: 3 times (at baseline, at 3 months, at 6 months)	I: 1 cognitive, behavioral; O: not applicable	outcome evaluation	self-reported appropriate respiratory protective equipment, occupational health knowledge (+), attitude (+), and practice e.g., participation in occupational health check-ups (+)
[61]	“Thrive at Work”: program focused on mental health, musculoskeletal health and a healthy lifestyle	multi-focused comprehensive program	6 months: 5 times (at baseline, after randomization, after 3 months, after 6 months and one year since intervention)	I: cognitive; behavioral; physiological; O: financial; corporate culture and strategy	outcome evaluation	sickness absence (+), health and safety compliance of workers (+), productivity and profit of the company (+), culture change in SMSs, happiness of workers, a fitter and more resilient workplace
[62]	Mental health promotion at the workplace, provision of mental support, individual and group talks, stress management training, personal development plans	health education and personalized counseling; coping with health problems and skills development	less than 3 months: 3 times (at baseline, at one month, at four months)	I: behavioral, psychological; O: corporate culture and strategy	outcome evaluation	empowerment (0), resilience, palliative behavior (+), determinants of four coping strategies of mental health (+), quality of life, and life satisfaction (+), unjustified worrying (−)
[63]	“Total Work Health ^®^”: occupational health and safety, employee safety	environmental and safety workplace intervention	no information	I: not applicable; O: corporate culture and strategy	process evaluation	smoke-free workplaces, cell phone use, personal protective equipment (PPE), equipment maintenance, flexible schedules, smoking cessation, weight management, physical activity, environmental changes e.g., installing bike racks, providing fitness equipment on site
[64]	“WHPOW”: health promotion of senior workers	environmental and safety workplace intervention, coping with health problems and skills development	no data indicated	I: cognitive, behavioral; O: corporate culture and strategy, financial	structure and process evaluation	transfer of knowledge, experience, ideas, and skills from older to younger workers, promoting the employment of older workers and increasing job retention among pre-retirement workers, work climate and attitudes toward older workers, fighting discrimination and exclusion, reducing the gender gap
[25]	Workplace wellness program	physical activity & fitness intervention	no data indicated	I: not applicable; O: corporate culture and strategy	structure and process evaluation	corporate culture and strategy of the small business organization; employees oriented towards more effective, viable and thriving wellness programs
[45]	Comprehensive integrated program, focused on working conditions, telephone health coaching and web-based resources that included integrated messages on back pain, worksite-wide events for ergonomic and health promotion practices	multi-focused comprehensive program	no data indicated	I: physiological, behavioral, cognitive; O: corporate culture and strategy, financial	structure, process and outcome evaluation	jointly predicted lower back pain (+), sleep (+), physical activity (+); employee-rated health culture and safety culture (+), self-reported back pain (−), safety hazards (-); organizational resources—measured by the CDC Worksite Health Scorecard: related to organizational support (+), physical activity (+) and nutrition (+)
[65]	Worksite fitness program, facilities and exercises	physical activity & fitness intervention	no data indicated	I: behavioral; O: corporate culture and strategy	process evaluation	social support and worksite environment (health promotion policy and equipment) affect employee participation in the program (+)
[85]	WHP program intervention including health education, diet education, physical fitness classes, smoking cessation classes, a smoke-free workplace	multi-focused comprehensive program	5 years	I: cognitive, behavioral; O: corporate culture and strategy	structure, process, and outcome evaluation	awareness of health, diet, physical activity, and smoking (+), using external resources and medical consultation (+), follow-up rates of the abnormal results of annual health examinations (+), the announcement of regulations (+), creating budgets specifically for health promotion and tobacco hazard control to improve employees’ physical and mental health conditions (+)
[67]	Programs including prevention and treatment of musculoskeletal disorder, promotion of physical activity, intervention in cases of chronic illness and cardiovascular risk factors; and a return-to-work program following injury, sickness or accident	multi-focused comprehensive program	long-term (several years)	I: cognitive, behavioral, physiological; O: corporate culture and strategy, financial	structure and process evaluation	the reduction of errors, increased safety, and performance of the person—machine—environment system, development of healthy lifestyle habits in the community, physical activity program as a strategy for prevention and health promotion for employees and their families (+)
[68]	“Participatory Action-Oriented Training [PAOT]”; program focused at improving health and safety at work, organizational and the individual level intervention, conducted to reduce work-related stress	multi-focused comprehensive program	2 months: 2 times (pre- and post-test)	I: cognitive, psychological, behavioral, physiological; O: corporate culture and strategy	outcome evaluation	blue-collar workers: stress (-), physical environment (+), occupational climate (+), job demands (+), job control (+), interpersonal conflicts (-), organizational system (+), and lack of rewards (-) white-collar: worker stress (0), physical environment (0) and occupational climate (+); job demands (0), job control (0), interpersonal conflicts (0), organizational system (0), and lack of rewards (0)
[69]	Intervention consists of engaging workplace-based ‘business champions’, integration, formalization and embedding in organizational environments by means of training and workshops, pedometer challenges and holistic therapy sessions	multi-focused comprehensive program	3 years: 1 time after intervention	I: cognitive, psychological, O: corporate culture and strategy	process evaluation	confidence building, capacity building and system change at individual and organizational levels, individual outcome knowledge improvement and wellbeing of employees; participatory approaches within interventions is a facilitator of the organizational culture (+)
[70]	Participatory Action-Oriented Training [PAOT], improving health and safety at work	multi-focused comprehensive program	1 year: 2 times (pre- and post-test)	I: not applicable; O: corporate culture and strategy, financial	outcome evaluation	improvements among the intervention factories in terms of work environment (+), number of improvements and health costs (+), productivity of civil engineering, metal, garment, and rice mill industries in the intervention group (+)
[71]	“Health Promotion Enterprise Program” psychosocial interventions, mental health promotion, provision of health services to people with mental illness, and professional skills training	multi-focused comprehensive program	30 months: 2 times (pre- and post-test)	I: psychological, behavioral; O: financial	outcome evaluation	participants’ ability to work (+), their sense of control over their jobs (+), ability to meet the mental demands of work (+), job stress levels (-) probability of absenteeism related to depression (-)
[72]	“Workplace Activator“ program promoting PA including access to a web portal with information on the benefits of PA and information on how to begin exercising, 3 months free gym membership, a free pedometer, challenges	physical activity & fitness intervention	6 months: 2 times (at baseline and after 6 months)	I: behavioral, physiological cognitive, psychological; O: corporate culture and strategy, financial	outcome evaluation	PA level and awareness (+), BMI (-), absenteeism (0), perceived social support for PA from friends (+), perceived social support for PA from family (0), after 6 months: physical activity (+), general health rating (+), satisfaction with life (+) and positive mood states (+), perceived stress (-), negative mood states (-) and presentism (-), absenteeism (0)
[73]	WHPP program consisting of four courses connected with lifestyle, Internet and printed material based	health education and personalized counseling	2–3-month: 2 times (at baseline and after 1 year)	I: behavioral, physiological; O: not applicable	outcome evaluation	change of lifestyle (+), overall prevalence of cardiovascular risk (-), 10% 10-year risk trend (-)
[74]	Identification of the enablers and barriers to introducing workplace health-promotion programs for SMEs	environmental and safety workplace intervention	no data indicated	I: not applicable; O: corporate culture and strategy, financial	process evaluation	factors determining the implementation of the program: an internal health champion/coordinator, resources, time, and the longevity of the external support funded by a government initiative
[75]	Worksite program consisted of physical fitness exercise for the occupational environment, aerobic exercise and stretching	physical activity & fitness intervention	3 months: 2 times (pre- and post-test)	I: behavioral, physiological, psychological; O: not applicable	outcome evaluation	weight (+), blood pressure (+), resting heart rate (+), waistline (+), BMI (+), front and back trunk flexibility (+), abdominal muscle durability (+) and back muscle strength (+), musculoskeletal disorders (+), cardiovascular risk factors (+), overall health (+)
[76]	Physical fitness program for small and medium-sized enterprises	physical activity & fitness intervention	no data indicated	I: not applicable; O: corporate culture and strategy, financial	structure and process evaluation	factors determining the realization of the strategy for workplace health promotion: ecological approach within the policy of the company, meaningful engagement by managers; protection from harm and opportunities for health improvement and affording protection for the viability and reputation of the business
[77]	Intervention using the Mental Health Action Checklist (list consisting of: sharing work planning, work time and organization, ergonomic work methods, workplace environments, mutual support at work, and preparedness and care) on reducing job stressors and psychological distress	multi-focused comprehensive program	6 months: 2 times (pre- and post-test)	I: psychological, behavioral, cognitive; O: corporate culture and strategy	structure, process, and outcome evaluation	reduction of job stressors (+) and psychological distress (+), skill underutilization (+), supervisor and coworker support (+), and job satisfaction, degree of worker participation and implementation of planned actions heavily influenced the intervention effect
[78]	“Inclusive Working Life“ program, reducing sickness absenteeism, promoting an early return to work, preventing early retirement, and promoting employment of functionally impaired persons	multi-focused comprehensive program	2 years: 2 times (pre- and post-test)	I: behavioral; O: finance	structure, process, outcome evaluation	sickness absenteeism (0), use of early retirement (+) and disability retirement (+), good cooperation with the occupational health service and the empowerment and involvement of the employees is associated with a low sickness absence rate
[79]	Empowerment model for workplace health promotion. The model consists of three tools: an action checklist, an information guidebook, and a book of good practices	multi-focused comprehensive program	1 year, information not clear	I: cognitive, behavioral; O: corporate culture and strategy	process evaluation	empowerment and participatory and action-oriented process of implementation WHPI in SMSs; WHP as part of organizational culture
[80]	“PACE’s Triangle of Prevention” health and safety, a comprehensive training program, effective participation, accident investigation and prevention	environmental and safety workplace intervention; health education and personalized counseling	no data indicated	I: not applicable; O: corporate culture and strategy	process evaluation	organizational culture, implementation of safety system
[81]	“Work Improvement in small Enterprises” program, improving the workplace environment, reducing the local muscle workloads, and preventing work-related muscle-skeletal disorder	environmental and safety workplace intervention	3 years: 3 times, at baseline, after implementation and one year after implementation	I: physiological; O: corporate culture and strategy	outcome evaluation	improvement the workplace environment (using the right tools, improving lighting conditions), health outcomes reducing the local muscle workloads and work-related muscle-skeletal disorder
[82]	as above	environmental and safety workplace intervention	2 years; follow up	I: not applicable; O: corporate culture and strategy, financial	outcome evaluation	frequency of occupational accidents (-); conditions of work (+) and working hours (+)
[24]	The intervention focused on the diagnosis of occupational health; assessment measures and measures for health-promoting work organization and job design	environmental and safety workplace intervention, coping with health problems and skills development	not applicable	I^:^ not applicable; O: corporate culture and strategy, financial	process evaluation	Factors determining the results of health promotion programs for enterprises were knowledge and attitude, support of external institutions in the process of implementation of WHP
[83].	pro-health education, coronary heart disease or musculoskeletal disorders	health education and personalized counseling	12 months	I: cognitive; O: not applicable	outcome evaluation	health promotion knowledge (+) attitude (+), subjective assessment of the usefulness of advice (+)
[84]	3 Programs: Work Improvementin Neighborhood Development Program, an action-oriented training program for trade unions. Goal: improve the workplace environment, reduce the local muscle workloads, and prevent work-related muscle-skeletal disorders	multi-focused comprehensive program	two weeks	I: not applicable; O: corporate culture and strategy, financial	outcome evaluation	improvement of ergonomics and working conditions of various groups of employees (+), number of accidents (-), working hours (+), occupational cost (-)

* According to the division from Table 3; ** (+)—improvement or favorable effect; (−) deterioration or unfavorable effect; (0)—no significant effect. Source: author’s compilation, *n* = 29.

**Table 4:** The evaluation criteria of strong evidence data confirming the effectiveness of WHPIs from an empirical review.

Studies	1.	2.	3.	4.	5.	6.	7.	8.
[59]	Y	Y	Y, after 1 year, after 2 and 3 years	Y	N	Y	Y	N
[60]	Y	Y	Y, at 6 months	Y	N	Y	Y	Y
[61]	Y	Y	Y, after 1 year	Y	N	Y	Y	Y
[62]	Y	Y	Y, after four months		N	Y	Y	N
[63]	N	ND	ND	Y	Y	Y	Y	N
[25]	N	ND	ND	N	Y	Y	Y	N
[45]	Y	ND	ND	Y	Y	Y	Y	N
[65]	N	ND	ND	Y	Y	Y	N	N
[66]	Y	ND	ND	Y	Y	Y	Y	N
[67]	ND	ND	ND	ND	Y	Y	Y	N
[68]	N	Y	N	Y	N	Y	Y	N
[70]	Y	Y	N	Y	N	Y	Y	Y
[71]	N	Y	N	Y	N	Y	Y	Y
[72]	N	Y	N	Y	N	Y	Y	Y
[73]	N	Y	N	Y	N	Y	N	N
[74]	N	ND	ND	N	Y	N	Y	Y
[75]	N	Y	N	Y	N	Y	Y	Y
[76]	N	ND	ND	N	Y	Y	Y	Y
[77]	N	Y	N	Y	Y	Y	Y	N
[78]	N	Y	N	Y	Y	Y	Y	Y
[79]	Y	ND	ND	Y	Y	Y	Y	N
[80]	N	ND	ND	N	Y	Y	Y	N
[81]	N	Y	Y, after 1 year	Y	N	Y	Y	Y
[82]	N	ND	ND	Y	N	Y	Y	Y
[24]	N	ND	ND	N	Y	Y	N	N
[83]	N	ND	ND	N	N	Y	Y	N
[84]	N	ND	ND	N	N	Y	Y	N

1. Were quasi-experimental methods or randomly controlled cluster trials or cross-sectorial studies used in the study to confirm the effectiveness of WHPI; 2. Was the WHPI effectiveness measurement carried out at least twice (at baseline and after intervention); 3. Was the WHPI effectiveness measurement repeated after a certain period of time after the end of the intervention to check the durability of its effects? After what period of time; 4. Were objectified research tools used in the study? 5. When assessing the effectiveness of the WHPI, was the structure or process examined in addition to the outcomes as well? 6. Was the WHPI effect to modify unhealthy habits and improve the risk profile of employees, especially the highest risk groups; 7. Was the WHPI effect an organizational change; 8. Did the WHPI result in financial savings for the organization; Y—yes; N—no; ND—no data indicated, *n* = 27.
